# Editorial: Novel Frontiers in Cancer Metastasis

**DOI:** 10.1007/s10585-022-10149-8

**Published:** 2022-02-12

**Authors:** Stanley P. Leong, Jonathan S. Zager

**Affiliations:** 1 California Pacific Medical Center and Research Institute, San Francisco, CA USA; 2 University of California San Francisco School of Medicine, San Francisco, CA USA; 3 Department of Cutaneous Oncology, Moffitt Cancer Center, Tampa, FL USA; 4 Morsani College of Medicine, University of South Florida, Tampa, USA

The 8th International Cancer Metastasis Congress being held between October 25–27, 2019 in San Francisco (www.cancermetastasis.org) addressed four major themes including cancer heterogeneity, cancer progression, mechanisms of metastasis through the lymphatic versus blood vessels and cancer therapy. These four major topics are represented in 23 review articles which are generated from the plenary presentations being delivered by multiple speakers. The content of these review articles is further discussed in the ensuing commentary on Introduction to this Special Issue: Novel Frontiers in Cancer Metastasis.

Cancer heterogeneity arising from genomic alterations or mutations has been firmly established on a molecular level. Within a cancer cell population, heterogenous clones exist which may have different metastatic potentials. The cancer microenvironment, consisting of stromal cells including immune cells, cancer-associated fibroblasts, endothelial cells and pericytes, may modulate the progression of cancer clones through production of cytokines to favor or inhibit cancer growth. Further, cancer cells may secrete autocrine cytokines to enhance their growth. Thus, the cancer microenvironment may act like a selective force similar to Darwinian “natural selection” to sort out the clones with the ability to spread from the primary site to the distant sites, according to Paget’s seed and soil hypothesis for cancer spread. The molecular mechanisms of cancer metastasis whether the initial pathway for cancer spread is through lymphatic or blood vessels, or via both pathways need to be further defined. These potential biomarkers of cancer metastasis will not only enable us to understand cancer spread on a molecular level but also allow us to develop therapeutic strategies in blocking the process of cancer metastasis.

We appreciate our sponsors and exhibitors who have made this Congress a success. They are acknowledged in our website (www.cancermetastasis.org). We are grateful to the Editorial Board of the Clinical and Experimental Metastasis to allow us to publish the presentations of the 8th International Cancer Metastasis Congress in this Special Issue. We are indebted to all the presenters and authors, who have summarized their presentations as review articles, which are separately published online and have been combined into this Special Issue, Novel Frontiers in Cancer Metastasis [1].

## Data Availability

Data sharing not applicable to this article as no datasets were generated or analyzed during the current commentary.
